# Percutaneous pedicle screw fixation combined with transforaminal endoscopic spinal canal decompression for the treatment of thoracolumbar burst fracture with severe neurologic deficit: A case report

**DOI:** 10.1097/MD.0000000000020276

**Published:** 2020-05-22

**Authors:** Zhangheng Huang, Chuan Hu, Yuexin Tong, Zhiyi Fan, Kewen Liu, Binbin Yang, Chengliang Zhao

**Affiliations:** a Department of Spine Surgery, Affiliated Hospital of Chengde Medical University, Shuangqiao District, Chengde, Hebei Province; b Department of Orthopedic Surgery, The Affiliated Hospital of Qingdao University, Shinan District, Qingdao, Shandong Province; c Department of Oncology, Ruian People's Hospital (Third Affiliated Hospital of Wenzhou Medical University), Wenzhou, Zhejiang Province, China.

**Keywords:** percutaneous pedicle screw fixation, thoracolumbar burst fracture, transforaminal endoscopic spinal canal decompression

## Abstract

**Rationale:**

The most common fractures of the spine are associated with the thoracolumbar junction (T10–L2). And burst fractures make up 15% of all traumatic thoracolumbar fractures, which are often accompanied by neurological deficits and require open surgeries. Common surgeries include either anterior, posterior or a combination of these approaches. Here, we report the first attempt to treat thoracolumbar burst fracture (TLBF) with severe neurologic deficits by percutaneous pedicle screw fixation (PPSF) and transforaminal endoscopic spinal canal decompression (TESCD).

**Patient concerns:**

A 46-year-old Chinese woman suffered from severe lower back pain with grade 0 muscle strength of lower limbs, without any sensory function below the injury level, with an inability to urinate or defecate after a motor vehicle accident. Imaging studies confirmed that she had Magerl type A 3.2 L1 burst fracture.

**Diagnoses:**

Burst fracture at L1.

**Interventions:**

The patient underwent PPSF at the level of T12 to L2, but her neurological function did not fully recover after the operation. One week after the injury, we performed TESCD on her.

**Outcomes:**

There was an immediate improvement in her neurological function in just 1 day after 2-stage operation. During the 6-month follow-up period, her neurological functions gradually recovered, and she was able to defecate and urinate. At the last follow-up visit, her spinal cord function was assessed to be at Frankel grade D.

**Lessons:**

PPSF plus TESCD can achieve complete spinal cord decompression, promote neurological recovery, and is therefore an effective method for the treating lumbar burst fractures with severe neurologic deficits.

## 1 Introduction

Burst fractures are common in the thoracolumbar fracture, and account for 21% to 58% of all spinal injuries.^[1,2]^ Thoracolumbar burst fracture (TLBF) is the most commonly the result of high-impact injury, such as motor vehicle accidents or falls from heights.^[3,4]^ In TLBF, the retropulsion of bone fragments into the spinal canal can cause neurological deficit.^[5]^ The optimal treatment for TLBF is controversial, despite the prevalence of large number of studies on this topic.^[6–8]^ Currently, it is widely believed that a neurological deficit is one of the absolute indications for immediate surgery to treat a thoracolumbar or lumbar burst fracture.^[9]^ The goal of surgery is to restore vertebral body height, correct kyphosis, decompress the spinal canal, prevent secondary spinal cord injury, and promote neurological recovery.^[10,11]^ Surgery can be performed via different approaches: anterior, posterior, or a combination of these two approaches.^[12]^ However, traditional open surgeries have the disadvantages of excessive blood loss, high incidence of complications, postoperative tissue ischemia, muscle atrophy, prolonged hospital stay, and long-term postoperative lower back pain.^[13–15]^ With the development in surgical techniques, more and more minimally invasive surgeries have been performed to treat burst fractures, which can achieve safe and effective results.^[16–18]^ Zhao et al reported the use of percutaneous pedicle screw fixation (PPSF) and transforaminal endoscopy (TE) for the treatment of L2 burst fracture with mild neurological damage and mid-sagittal canal diameter compression ratio (MSDCR) <50%.^[3]^ To the best of our knowledge, TLBF with MSDCR >50% and severe neurological deficits treated with PPSF and transforaminal endoscopic spinal canal decompression (TESCD) have not been reported. Here, we describe a case of lumbar burst fracture with preoperative Frankel grade A and MSDCR 70% treated with PPSF and TESCD.

## 2 Case report

This study was approved by the Ethics Committee and institutional Review Board of the Affiliated Hospital of Chengde Medical University.

**Figure 1:**
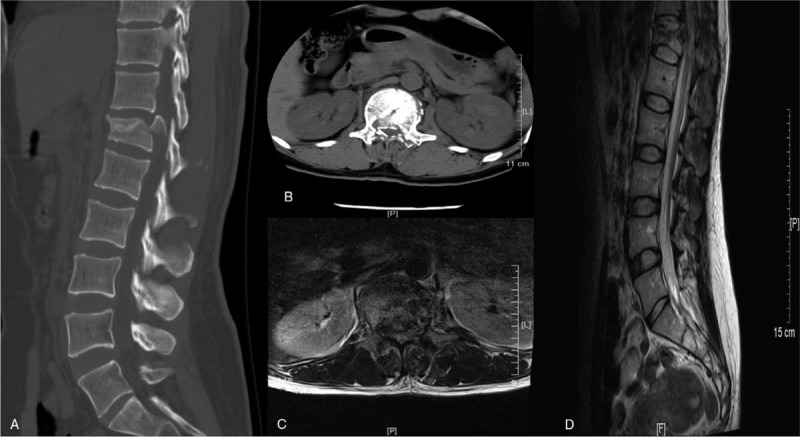
Imaging of the patient on admission. On the sagittal (A) and axial (B) computed tomography, the retropulsion of bone fragments into the spinal canal. Magnetic resonance imaging of T2-weighted image (C) and (D) also showed that bone fragments had retropulsed into the spinal canal and dural sac was severely compressed.

A 46-year-old Chinese woman visited our hospital 2 h after a motor vehicle accident. She had severe lower back pain, grade 0 muscle strength of lower limbs, no sensory function below the injury level, and an inability to urinate or defecate. The patient was alert but had no motor function below the groin level. Neurological examination showed that her muscle strength in the bilateral lower extremities was grade 0/5, with an obvious hypesthesia below the level of groin, impaired sensation of the perineum region, and absent voluntary anal sphincter contraction. Visual analog scale (VAS) score for back pain was 10. Computed tomography (CT) showed L1 burst fracture, and retropulsion of bone fragments into the spinal canal, with MSDCR 70.3% (Fig. 1). Magnetic resonance imaging (MRI) showed that bone fragments had retropulsed into the spinal canal and dural sac was severely compressed (Fig. 1). And the patient was diagnosed with a Magerl type A 3.2 L1 burst fracture. Given the patient's condition, emergency surgery was needed to prevent secondary spinal cord injury and induce neurologic recovery. We first performed PPSF to stabilize the spine, correct deformity, and achieve indirect decompression of the spinal canal via percutaneous reduction internal fixation.^[19–21]^ The specific procedure for PPSF was as follows: The patient was put under local infiltration anesthesia and in a prone position. The C-arm fluoroscope was used to determine the skin entry point. Infiltration anesthesia of the periosteum was achieved with the 0.5% lidocaine injection. The puncture needle was advanced into the vertebra via the vertebral pedicle guided with fluoroscopic imaging, followed by guide wire insertion. Suitable cannulated screws were inserted percutaneously along the guide wire and connected with a rod. The screws were tightened and fixed to realign the injured vertebra. The operating time is 1 h and the blood loss is about 80 mL. After the operation, the patient's VAS score decreased to 3 and muscle strength of the bilateral lower extremities improved to grade 1/5. She also had complete sensory function in her right limbs, alleviated sensory loss of the perineum region, and a recovered voluntary anal sphincter contraction.

**Figure 2:**
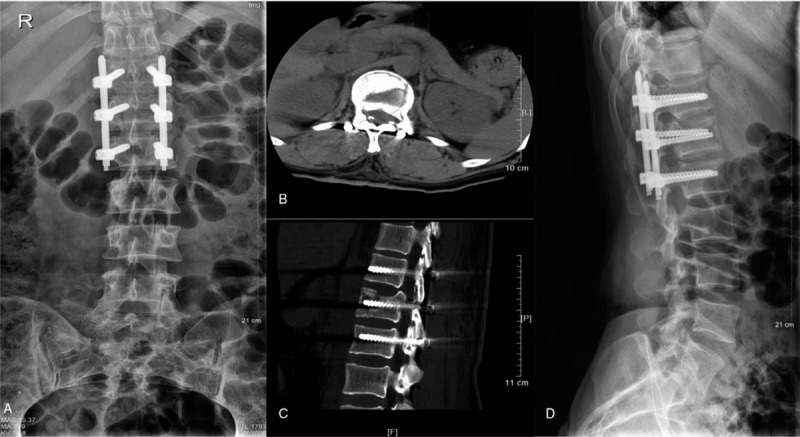
The images of the patient were re-examined on the first day after PPSF. The first postoperative radiograph showed satisfactory position of the internal fixation system (A and D). The axial (B) and sagittal (C) of the computed tomography showed the size of fracture fragments in the spinal canal decreased.

**Figure 3:**
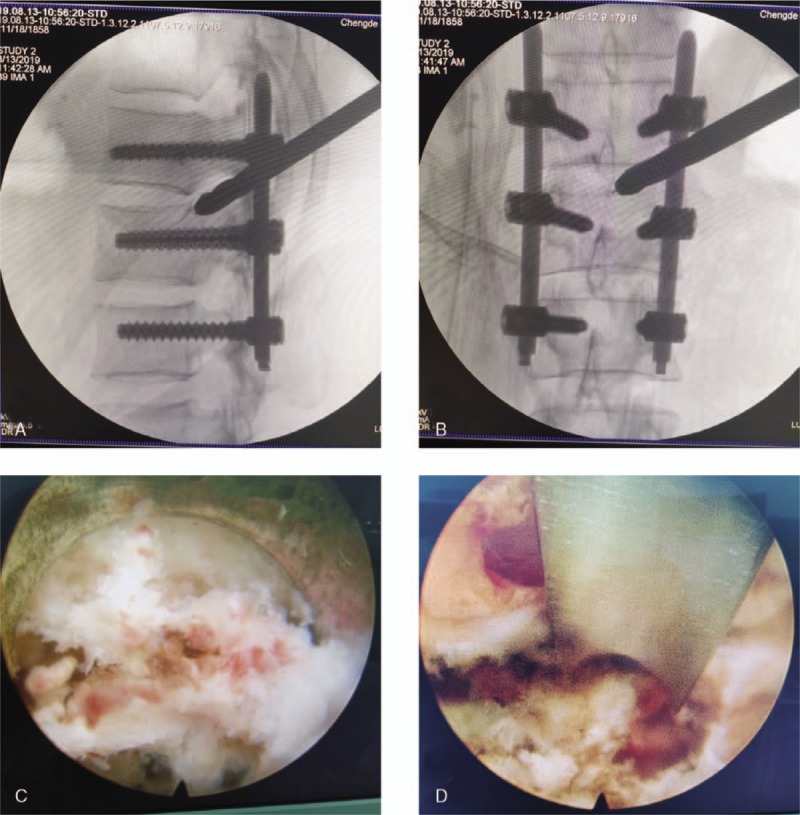
The distal end of the cannula was extended to the median part of the spinal canal (A) and the cannula tip reached the posterior–superior end of the L1 vertebra (B), as visualized by C-arm fluoroscopy. The remaining fracture fragments in the spinal canal can be seen under the transforaminal endoscope (C and D).

**Figure 4:**
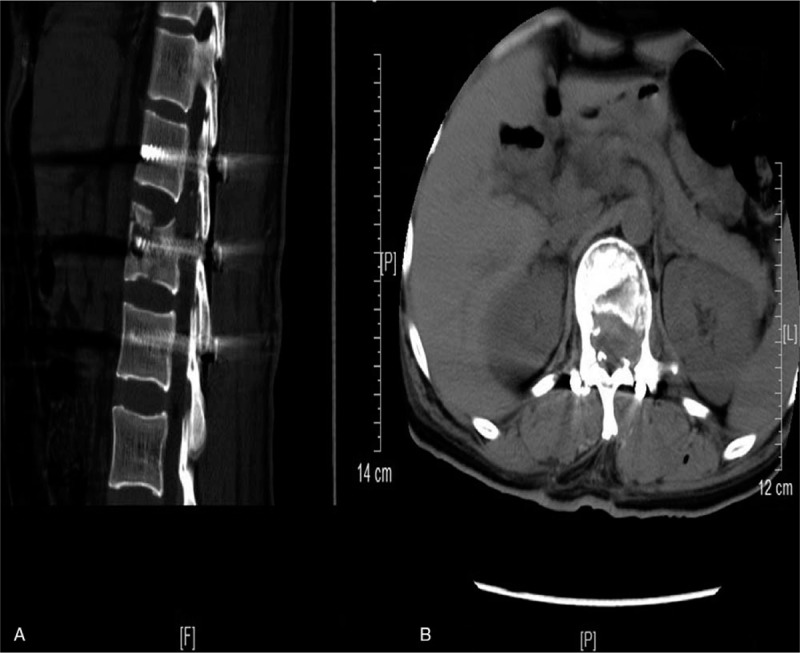
The images of the patients were re-examined on the 3rd day after the second operation. The sagittal (A) and axial (B) of computed tomography showed no residual bone fragments in the spinal canal and complete decompression of the spinal cord.

The size of fracture fragments in the spinal canal decreased (Fig. 2), but partial neurologic deficit remained. Therefore, on the 5th day after PPSF, we performed TESCD on this patient to decompress the spinal canal. The patient was put in a right lateral decubitus position with cushions placed at her waist. Fluoroscopic imaging was used to determine the entry point on the skin, and 0.8% lidocaine was injected to infiltratively anesthetize the subcutaneous tissues, fascia and joint capsule. Guided by a C-arm fluoroscope, a 16-gauge needle was inserted directly into the tip of the superior articular process. The puncture tract was dilated and foraminaplasty was performed using bone drills with diameters of 4, 6, and 8 mm. We inserted a working cannula into the dilated tract through the foramen and extended the distal end of the cannula to the median part of the spinal canal until the tip of cannula reached the posterior–superior end of the L1 vertebra (Fig. 3). During the operation, we payed specific attention to the patient's reaction to avoid accidental nerve injury. Under the fluoroscope, we decompressed the dural sac and nerve roots by removing fractured fragments of the vertebral body, as well as damaged annulus fibrosus and ligaments that were pressing against the spinal cord. Fluoroscopic imaging was used to confirm that there were no residuals left. One drainage tube was placed and the operation was completed. Total operating time was 1.5 h, and the blood loss was approximately 40 mL. Physical examination performed at day 1 after operation showed that the patient's muscle strength of lower limbs improved to 2/5. The patient also had significantly improved sensation of the perineum region and left lower limb. At day 3 after operation, CT and MRI revealed no residual bone fragments in the spinal canal and complete decompression of the spinal cord, with MSDCR 0% (Fig. 4). The Cobb angle (CA) decreased from 13.6° to 2.4°, and the vertebral wedge angle reduced from 7.0° to 4.9°. The vertebral body compression ratio dropped from 28.1% to 1.6%. The specific outcome assessment parameters (pre-operation and the post-operation) were shown in Table 1. Two weeks after TESCD, her muscle strength in the lower limbs increased to grade 3 with only minor numbness. During the 6-month follow-up period, the patient had a VAS score of 0 for back pain. Most of her sensory and motor functions recovered, and she could walk a short distance with a slow gait.

**Table 1:**
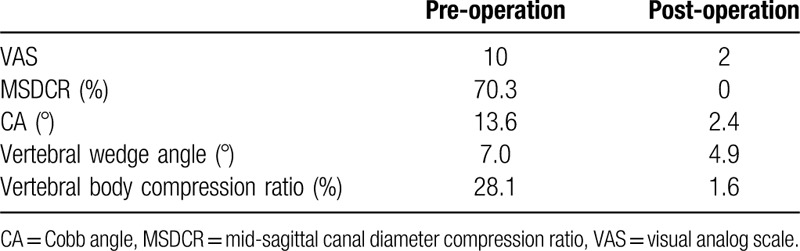
Specific outcome assessment parameters (pre-operation and the post-operation).

## 3 Discussion

Here, we present the first use of PPSE and TESCD in combination for treating TLBF with MSDCR >50% and severe neurological deficits. Zhao et al reported the use of PPSF and TE in the treatment of L2 burst fracture with mild neurological damage and MSDCR <50%, which achieved excellent efficacy during the 2-year follow-up. They suggested that PPSF and TE are feasible to treat burst fractures with transverse and sagittal diameter of compression mass no >15 and 10 mm, respectively, and with the compression area no >50% in radiological evaluation.^[3]^ In this study, we modified the surgical techniques for PPSF and TESCD, used them for treating a patient with burst fracture and MSDCR >50%, and achieved excellent results. The patient's spinal cord function improved to grade D, 2 weeks after operation. She also had completely recovered the ability to defecate and urinate, had MSDCR score of 0, and VBCR 1.6% after the operation. All these results proved the favorable efficacy of the combined use of PPSF and TESCD.

Currently, surgical options for TLBF with severe neurological deficits include posterior pedicle screw fixation combined with posterior decompression, anterior decompression combined with anterior fixation, anterior decompression and fixation combined with posterior fixation.^[12]^ The anterior approach has the advantages of direct decompression and anterior column support. However, there is a risk of secondary nerve damage when removing large bone fragments in the spinal cord via this approach. Also, the anterior approach is disadvantaged by large trauma and higher incidence of complications.^[22]^ The posterior approach causes less trauma, but it is less effective in relieving pressure on the spinal cord, and is associated with a higher risk of procedure-related secondary nerve damage, compared with the anterior approach .^[11,12,21]^ In contrast, PPSF can achieve the same fixation effect as the open posterior approach, and with fewer approach-related complications.^[23,24]^ In TLBF, neurological deficits are usually caused by compression of the ventral surface of the spinal cord. The anterior approach can provide more direct decompression than the posterior approach and may be more conducive for the recovery of neurological function. However, the combined anterior–posterior surgery without an increasing improvement in neurological function recovery when compared with the anterior surgery or posterior surgery. For TLBF patients with neurological deficits, PPSF can obtain good indirect spinal canal decompression, and some cases may obtain good neurological function recovery without further decompression. However, according to the recovery of neurological function after PPSF and the spinal canal decompression displayed by CT, we decided to further carry out TESCD on the patient. Bleeding at the fracture site may affect the field of view under the microscope, which in turn affects the surgical procedure and efficacy. Therefore, we did not perform TESCD prematurely, but performed surgery on the fifth day postoperatively. Theoretically, spinal canal decompression should be completed as soon as the condition permits, because the long interval between two operations may affect the recovery of neurological function. Therefore, in the future, according to the patient's condition, we can consider to shorten the interval between TESCD and PPSF, or even performed simultaneously. TESCD allows direct spinal cord decompression just like the anterior approach, but with significantly less surgical trauma, which is essential for the recovery of neurological. By using the method of three nerve root decompression, the fractured bones in the ventral dural sac and broken disc tissues can be fully removed, and the contralateral pedicle can be decompressed to the inner edge of the pedicle. Additionally, it offers maximum safety for the patients. As this procedure is performed under local infiltration anesthesia, it is possible to maintain effective intraoperative communication with patients. But for the open surgery, general anesthesia is required, and the risk of secondary spinal cord and nerve injury is high as surgeons receive no real-time feedback from patients who are unconscious during the operation.

With progress in surgical techniques, minimally invasive surgeries have become more popular, and can be applied to treat many different conditions. It is even possible that contraindications become indications for these procedures. In this report, we preformed PPSF and TESCD to treat a patient with L1 burst fracture, MSDCR >70%, and severe neurological deficit. We extended indications for such procedures and proved that PPSF and TESCD can treat TLBF with severe neurological deficits. Of course, the understanding and skillful application of PPSF technique and three nerve root decompression technique are the main factors that determine the indication.

However, the combined use of PPSF and TESCD in the treatment of burst fractures is not without limitations. First, minimally invasive surgery (i.e., PPSF combined with TESCD) requires clinicians to be proficient in PPSF and TESCD. Among them, decompression of the spinal canal requires unilateral approach through the intervertebral foramen to achieve bilateral decompression, which is difficult. Second, although the relationship between the neurological recovery and the timing of decompression is still inconclusive, there is a possibility that the second phase of TESCD will miss the optimal period of spinal cord decompression. Lastly, according to our experience, the indication for minimally invasive surgery (i.e., PPSF combined with TESCD) is lumbar burst fractures with neurological deficits, but further practice and research are still needed to prove it.

## 4 Conclusions

The key to the recovery of neurological function is complete spinal cord decompression for patients with severe lumbar burst fractures. And a minimally invasive surgery (i.e., PPSF combined with TESCD) is effective for such purpose.

## Acknowledgments

We would like to thank all the staff in Department of Spine Surgery, Affiliated Hospital of Chengde Medical University for their contribution on our research.

## Author contributions

**Conceptualization:** Zhangheng Huang, Chengliang Zhao.

**Data curation:** Zhangheng Huang.

**Formal analysis:** Chuan Hu.

**Investigation:** Yuexin Tong, Zhiyi Fan.

**Methodology:** Zhangheng Huang.

**Project administration:** Kewen Liu, Binbin Yang.

**Supervision:** Zhangheng Huang, Chuan Hu, Chengliang Zhao.

**Writing – original draft:** Zhangheng Huang.

**Writing – review & editing:** Zhangheng Huang.
